# Supplementary material for: Highly Unsaturated Platinum and Palladium Carbenes PtC3 and PdC3 Isolated and Characterized in the Gas Phase
Source: Angew Chem Weinheim Bergstr Ger. 2016 Feb 15;128(11):3832–5. doi: 10.1002/ange.201511646 (PMC4950143; doi:10.1002/ange.201511646)

Angewandte Chemie
Eine Zeitschrift der Gesellschaft Deutscher Chemiker

Supporting Information

Highly Unsaturated Platinum and Palladium Carbenes PtC_3 and PdC_3 Isolated and Characterized in the Gas Phase

Dror M. Bittner, Daniel P. Zaleski, David P. Tew, Nicholas R. Walker, and Anthony C. Legon**

ange_201511646_sm_miscellaneous_information.pdf

Supplementary Data

Table of Contents

p.2 : Supplementary Data Table 1 – Summary of results of fits of spectroscopic parameters of PdC_3 and PtC_3.

p.3 : Supplementary Data Table 2 -*Ab initio* calculated harmonic wavenumbers of PdC_3 and PtC_3.

p.3 : Supplementary Data Table 3 - CCSD(T)(F12*) Calculation of dissociation energies of PdC_3 and PtC_3.

p.4 onwards: Supplementary Data Tables 4-36 - Measured transition frequencies and residuals for PdC_3 and PtC_3 presented in the following order;
p.4: $^{104}Pd^{12}C^{12}C^{12}C$
p.4: $^{105}Pd^{12}C^{12}C^{12}C$
p.4: $^{106}Pd^{12}C^{12}C^{12}C$
p.5: $^{106}Pd^{13}C^{13}C^{13}C$
p.5: $^{108}Pd^{12}C^{12}C^{12}C$
p.5: $^{108}Pd^{13}C^{13}C^{13}C$
p.5: $^{110}Pd^{12}C^{12}C^{12}C$
p.5: $^{194}Pt^{12}C^{12}C^{12}C$
p.5: $^{194}Pt^{13}C^{13}C^{13}C$
p.6: $^{194}Pt^{12}C^{12}C^{13}C$
p.6: $^{194}Pt^{12}C^{13}C^{12}C$
p.6: $^{194}Pt^{13}C^{12}C^{12}C$
p.6: $^{194}Pt^{13}C^{13}C^{12}C$
p.6: $^{194}Pt^{13}C^{12}C^{13}C$
p.6: $^{194}Pt^{12}C^{13}C^{13}C$
p.7: $^{195}Pt^{12}C^{12}C^{12}C$
p.7: $^{195}Pt^{13}C^{13}C^{13}C$
p.7: $^{195}Pt^{12}C^{12}C^{13}C$
p.7: $^{195}Pt^{12}C^{13}C^{12}C$
p.7: $^{195}Pt^{13}C^{12}C^{12}C$
p.7: $^{195}Pt^{13}C^{13}C^{12}C$
p.8: $^{195}Pt^{13}C^{12}C^{13}C$
p.8: $^{195}Pt^{12}C^{13}C^{13}C$
p.8: $^{196}Pt^{12}C^{12}C^{12}C$
p.8: $^{196}Pt^{13}C^{13}C^{13}C$
p.8: $^{196}Pt^{12}C^{12}C^{13}C$
p.8: $^{196}Pt^{12}C^{13}C^{12}C$
p.9: $^{196}Pt^{13}C^{12}C^{12}C$
p.9: $^{196}Pt^{13}C^{13}C^{12}C$
p.9: $^{196}Pt^{13}C^{12}C^{13}C$
p.9: $^{196}Pt^{12}C^{13}C^{13}C$
p.9: $^{198}Pt^{12}C^{12}C^{12}C$
p.9: $^{198}Pt^{13}C^{13}C^{13}C$

p.10: Supplementary Data Figure 1 - Frontier orbital energy level diagram for PtC_3 calculated at the HF/aug-cc-pVTZ level of theory.

Supplementary Data Table 1 – Summary of results of fits of spectroscopic parameters of PdC_3 and PtC_3.

	B_0 / MHz	$[D_J \times 10^2]$ / kHz	N[a]	σ_{rms}[a] / kHz
$^{104}Pd^{12}C^{12}C^{12}C$	1709.52303(75)	[9.6][b]	4	11.1
$^{105}Pd^{12}C^{12}C^{12}C$	1705.8926(24)	[9.6][c]	9	14.5
$^{106}Pd^{12}C^{12}C^{12}C$	1702.33446(43)	[9.5]	4	6.3
$^{106}Pd^{13}C^{13}C^{13}C$	1599.5465[d]	[8.4]	1	-
$^{108}Pd^{12}C^{12}C^{12}C$	1695.40016(42)	[9.4]	4	6.2
$^{108}Pd^{13}C^{13}C^{13}C$	1592.65454(49)	[8.3]	2	6.3
$^{110}Pd^{12}C^{12}C^{12}C$	1688.70725(75)	[9.4]	4	11.0
$^{194}Pt^{12}C^{12}C^{12}C$	1592.94589(35)	[6.2]	3	5.0
$^{194}Pt^{13}C^{13}C^{13}C$	1486.74426(60)	[5.4]	4	11.1
$^{194}Pt^{12}C^{12}C^{13}C$	1522.47541(52)	[5.6]	3	9.1
$^{194}Pt^{12}C^{13}C^{12}C$	1560.77071(27)	[6.0]	2	3.4
$^{194}Pt^{13}C^{12}C^{12}C$	1585.00114(43)	[6.2]	3	6.1
$^{194}Pt^{13}C^{13}C^{12}C$	1553.27906(75)	[6.0]	3	10.6
$^{194}Pt^{13}C^{12}C^{13}C$	1515.40560(35)	[5.6]	3	6.2
$^{194}Pt^{12}C^{13}C^{13}C$	1493.42717(29)	[5.5]	3	5.0
$^{195}Pt^{12}C^{12}C^{12}C$	1591.82514(63)	[6.2]	3	9.0
$^{195}Pt^{13}C^{13}C^{13}C$	1485.62696(31)	[5.4]	4	5.7
$^{195}Pt^{12}C^{12}C^{13}C$	1521.3794(11)	[5.6]	4	19.8
$^{195}Pt^{12}C^{13}C^{12}C$	1559.645566(83)	[6.0]	2	10.6
$^{195}Pt^{13}C^{12}C^{12}C$	1583.86590(45)	[6.2]	3	6.4
$^{195}Pt^{13}C^{13}C^{12}C$	1552.13785[d]	[5.9]	1	-
$^{195}Pt^{13}C^{12}C^{13}C$	1514.29410(32)	[5.6]	3	5.6
$^{195}Pt^{12}C^{13}C^{13}C$	1492.322703(58)	[5.4]	3	10.1
$^{196}Pt^{12}C^{12}C^{12}C$	1590.71658(69)	[6.2]	3	9.8
$^{196}Pt^{13}C^{13}C^{13}C$	1484.52286(27)	[5.4]	3	4.7
$^{196}Pt^{12}C^{12}C^{13}C$	1520.29375(70)	[5.6]	3	12.2
$^{196}Pt^{12}C^{13}C^{12}C$	1558.53032(22)	[6.0]	2	2.8
$^{196}Pt^{13}C^{12}C^{12}C$	1582.74389(90)	[6.2]	3	12.7
$^{196}Pt^{13}C^{13}C^{12}C$	1551.0092(16)	[5.9]	2	20.7
$^{196}Pt^{13}C^{12}C^{13}C$	1513.1966(10)	[5.6]	2	16.1
$^{196}Pt^{12}C^{13}C^{13}C$	1491.23196(56)	[5.4]	3	9.8
$^{198}Pt^{12}C^{12}C^{12}C$	1588.53039(16)	[6.2]	3	2.2
$^{198}Pt^{13}C^{13}C^{13}C$	1482.34380(75)	[5.4]	3	13.1

a. N is the number of fitted transitions. σ_{rms} denotes the rms deviation of the fit.
b. All centrifugal distortion constants are fixed to results calculated *ab initio* performed using GAUSSIAN 09 package at the MP2 level of theory using the basis sets aug-cc-pVTZ on C atoms and aug-cc-pVTZ-PP with combination with the associated ECP on Pt and Pd.
c. ^{105}Pd has I = 5/2 yielding hyperfine splitting in measured rotational transitions. Nine hyperfine components were measured for the $^{105}Pd^{12}C^{12}C^{12}C$ isotopologue allowing a value of $\chi_{aa}(^{105}Pd)$ = 35.88(43) MHz to be fitted.
d. Parameter determined through measurement of only one transition so no statistical uncertainty can be associated with the result.

Supplementary Data Table 2 – Harmonic wavenumbers (ω_σ /c) computed at the CCSD(T)(F12*)/ACVTZ level of theory.

	(ω_σ /c) / cm^{-1}	
Vibrational mode	PdC_3	PtC_3
CCC bend	109	122
MCC bend	314	416
MC stretch	452	517
CCC sym. stretch	1342	1430
CCC asym. stretch	2086	2102

Supplementary Data Table 3 – CCSD(T)(F12*) Calculation of dissociation energies of PdC_3 and PtC_3.

	Energy (/ E_h)
PdC_3	–241.599
PtC_3	–233.472[a]
C_3	–114.028[b]
Pd(^{1}S)	–127.459
Pt(^{3}D) (uncorrected)	–119.266[a,c]
Pt(^{3}D) (corrected)	–119.285[a,c]
D_e ($PdC_3 \rightarrow Pd + C_3$)	0.112 = 295 kJ mol^{-1}
D_e ($PtC_3 \rightarrow Pt + C_3$)[d]	0.159 = 417 kJ mol^{-1}

[a] CABS singles not included.
[b] The energy of C_3 is calculated to be –114.026 E_h when CABS singles are not included.
[c] The “uncorrected” calculation for Pt(^{3}D) takes no account of spin-orbit coupling. The “corrected” calculation shows the energy after accounting for spin-orbit coupling quantified with reference to the experimental line spectrum of the neutral Pt atom as follows;

$^3D_3$ 0 cm^{-1}
$^3D_2$ 6567.5 cm^{-1}
$^3D_1$ 10131.9 cm^{-1}

retrieved from;
<http://physics.nist.gov/PhysRefData/Handbook/Tables/platinumtable5.htm>

The correction applied to the calculated result is thus;
$-((3 \times 10131.9) + (5 \times 6567.5) + (7 \times 0))/15 = -4215 \text{ cm}^{-1} = -0.0192 \text{ E}_h$

[d] Calculated with respect to the result for Pt(^{3}D) after correcting for spin-orbit coupling.

Supplementary Data Table 4: $^{104}Pd^{12}C^{12}C^{12}C$

$J' \rightarrow J''$	Observed Frequency / (MHz)	Calculated Frequency / (MHz)	Obs.– Calc. / (MHz)
$5 \rightarrow 4$	17095.1750	17095.1823	-0.0073
$4 \rightarrow 3$	13676.1750	13676.1597	0.0153
$3 \rightarrow 2$	10257.1250	10257.1278	-0.0028
$2 \rightarrow 1$	6838.0810	6838.0891	-0.0081

Supplementary Data Table 5: $^{105}Pd^{12}C^{12}C^{12}C$

$J' \rightarrow J''$ ($F' \rightarrow F''$)	Observed Frequency / (MHz)	Calculated Frequency / (MHz)	Obs.– Calc. / (MHz)
$5 \rightarrow 4$ ($4.5 \rightarrow 3.5$)	17059.4460	17059.4359	0.0101
$5 \rightarrow 4$ ($5.5 \rightarrow 4.5$)	17058.9500	17058.9435	0.0065
$5 \rightarrow 4$ ($7.5 \rightarrow 6.5$)	17058.6500	17058.6674	-0.0174
$5 \rightarrow 4$ ($6.5 \rightarrow 5.5$)	17058.5000	17058.5168	-0.0168
$4 \rightarrow 3$ ($4.5 \rightarrow 4.5$)	13647.3750	13647.3796	-0.0046
$4 \rightarrow 3$ ($4.5 \rightarrow 3.5$)	13647.3750	13647.3770	-0.0020
$4 \rightarrow 3$ ($6.5 \rightarrow 5.5$)	13646.8500	13646.8266	0.0234
$3 \rightarrow 2$ ($5.5 \rightarrow 4.5$)	10234.9000	10234.9049	-0.0049
$3 \rightarrow 2$ ($4.5 \rightarrow 3.5$)	10234.5750	10234.5630	0.0120

Supplementary Data Table 6: $^{106}Pd^{12}C^{12}C^{12}C$

$J' \rightarrow J''$	Observed Frequency / (MHz)	Calculated Frequency / (MHz)	Obs.– Calc. / (MHz)
$5 \rightarrow 4$	17023.3000	17023.2971	0.0029
$4 \rightarrow 3$	13618.6500	13618.6514	-0.0014
$3 \rightarrow 2$	10214.0000	10213.9965	0.0035
$2 \rightarrow 1$	6809.3250	6809.3348	-0.0098

Supplementary Data Table 7: $^{106}Pd^{13}C^{13}C^{13}C$

$J' \rightarrow J''$	Observed Frequency / (MHz)	Calculated Frequency / (MHz)	Obs.– Calc. / (MHz)
$5 \rightarrow 4$	15995.4230	15995.4230	0.0000

Supplementary Data Table 8: $^{108}Pd^{12}C^{12}C^{12}C$

$J' \rightarrow J''$	Observed Frequency / (MHz)	Calculated Frequency / (MHz)	Obs.– Calc. / (MHz)
$5 \rightarrow 4$	16953.9500	16953.9546	-0.0046
$4 \rightarrow 3$	13563.1750	13563.1772	-0.0022
$3 \rightarrow 2$	10172.4000	10172.3908	0.0092
$2 \rightarrow 1$	6781.6000	6781.5976	0.0024

Supplementary Data Table 9: $^{108}Pd^{13}C^{13}C^{13}C$

$J' \rightarrow J''$	Observed Frequency / (MHz)	Calculated Frequency / (MHz)	Obs.– Calc. / (MHz)
$5 \rightarrow 4$	15926.5000	15926.5039	-0.0039
$4 \rightarrow 3$	12741.2200	12741.2151	0.0049

Supplementary Data Table 10: $^{110}Pd^{12}C^{12}C^{12}C$

$J' \rightarrow J''$	Observed Frequency / (MHz)	Calculated Frequency / (MHz)	Obs.– Calc. / (MHz)
$5 \rightarrow 4$	16887.0230	16887.0255	-0.0025
$4 \rightarrow 3$	13509.6250	13509.6339	-0.0089
$3 \rightarrow 2$	10132.2500	10132.2333	0.0167
$2 \rightarrow 1$	6754.8250	6754.8260	-0.0010

Supplementary Data Table 11: $^{194}Pt^{12}C^{12}C^{12}C$

$J' \rightarrow J''$	Observed Frequency / (MHz)	Calculated Frequency / (MHz)	Obs.– Calc. / (MHz)
$5 \rightarrow 4$	15929.4250	15929.4409	–0.0159
$4 \rightarrow 3$	12743.5500	12743.5616	–0.0116
$3 \rightarrow 2$	9557.6750	9557.6764	–0.0014

Supplementary Data Table 12: $^{194}Pt^{13}C^{13}C^{13}C$

$J' \rightarrow J''$	Observed Frequency / (MHz)	Calculated Frequency / (MHz)	Obs.– Calc. / (MHz)
$6 \rightarrow 5$	17840.8750	17840.8845	-0.0095
$5 \rightarrow 4$	14867.4250	14867.4156	0.0094
$4 \rightarrow 3$	11893.9500	11893.9403	0.0097
$3 \rightarrow 2$	8920.4500	8920.4597	-0.0097

Supplementary Data Table 13: $^{194}Pt^{12}C^{12}C^{13}C$

$J' \rightarrow J''$	Observed Frequency / (MHz)	Calculated Frequency / (MHz)	Obs.– Calc. / (MHz)
$6 \rightarrow 5$	18269.6500	18269.6565	-0.0065
$5 \rightarrow 4$	15224.7250	15224.7261	-0.0011
$4 \rightarrow 3$	12179.8000	12179.7889	0.0111

Supplementary Data Table 14: $^{194}Pt^{12}C^{13}C^{12}C$

$J' \rightarrow J''$	Observed Frequency / (MHz)	Calculated Frequency / (MHz)	Obs.– Calc. / (MHz)
$5 \rightarrow 4$	15607.6750	15607.6771	-0.0021
$4 \rightarrow 3$	12486.1530	12486.1503	0.0027

Supplementary Data Table 15: $^{194}Pt^{13}C^{12}C^{12}C$

$J' \rightarrow J''$	Observed Frequency / (MHz)	Calculated Frequency / (MHz)	Obs.– Calc. / (MHz)
$5 \rightarrow 4$	15849.9750	15849.9804	-0.0054
$4 \rightarrow 3$	12680.0000	12679.9932	0.0068
$3 \rightarrow 2$	9510.0000	9510.0001	-0.0001

Supplementary Data Table 16: $^{194}Pt^{13}C^{13}C^{12}C$

$J' \rightarrow J''$	Observed Frequency / (MHz)	Calculated Frequency / (MHz)	Obs.– Calc. / (MHz)
$5 \rightarrow 4$	15532.7500	15532.7606	-0.0106
$4 \rightarrow 3$	12426.2250	12426.2171	0.0079
$3 \rightarrow 2$	9319.6750	9319.6679	0.0071

Supplementary Data Table 17: $^{194}Pt^{13}C^{12}C^{13}C$

$J' \rightarrow J''$	Observed Frequency / (MHz)	Calculated Frequency / (MHz)	Obs.– Calc. / (MHz)
$6 \rightarrow 5$	18184.8250	18184.8188	0.0062
$5 \rightarrow 4$	15154.0250	15154.0280	-0.0030
$4 \rightarrow 3$	12123.2250	12123.2305	-0.0055

Supplementary Data Table 18: $^{194}Pt^{12}C^{13}C^{13}C$

$J' \rightarrow J''$	Observed Frequency / (MHz)	Calculated Frequency / (MHz)	Obs.– Calc. / (MHz)
$6 \rightarrow 5$	17921.0760	17921.0786	-0.0026
$5 \rightarrow 4$	14934.2500	14934.2442	0.0058
$4 \rightarrow 3$	11947.4000	11947.4033	-0.0033

Supplementary Data Table 19: $^{195}Pt^{12}C^{12}C^{12}C$

$J' \rightarrow J''$	Observed Frequency / (MHz)	Calculated Frequency / (MHz)	Obs.– Calc. / (MHz)
$5 \rightarrow 4$	15918.2250	15918.2204	0.0046
$4 \rightarrow 3$	12734.5750	12734.5852	-0.0102
$3 \rightarrow 2$	9550.9500	9550.9441	0.0059

Supplementary Data Table 20: $^{195}Pt^{13}C^{13}C^{13}C$

$J' \rightarrow J''$	Observed Frequency / (MHz)	Calculated Frequency / (MHz)	Obs.– Calc. / (MHz)
$6 \rightarrow 5$	17827.4750	17827.4769	-0.0019
$5 \rightarrow 4$	14856.2500	14856.2426	0.0074
$4 \rightarrow 3$	11885.0000	11885.0019	-0.0019
$3 \rightarrow 2$	8913.7500	8913.7559	-0.0059

Supplementary Data Table 21: $^{195}Pt^{12}C^{12}C^{13}C$

$J' \rightarrow J''$	Observed Frequency / (MHz)	Calculated Frequency / (MHz)	Obs.– Calc. / (MHz)
$6 \rightarrow 5$	18256.5000	18256.5043	-0.0043
$5 \rightarrow 4$	15213.7500	15213.7659	-0.0159
$4 \rightarrow 3$	12171.0250	12171.0208	0.0042
$3 \rightarrow 2$	9128.3000	9128.2703	0.0297

Supplementary Data Table 22: $^{195}Pt^{12}C^{13}C^{12}C$

$J' \rightarrow J''$	Observed Frequency / (MHz)	Calculated Frequency / (MHz)	Obs.– Calc. / (MHz)
$5 \rightarrow 4$	15596.4250	15596.4257	-0.0007
$4 \rightarrow 3$	12477.1500	12477.1492	0.0008

Supplementary Data Table 23: $^{195}Pt^{13}C^{12}C^{12}C$

$J' \rightarrow J''$	Observed Frequency / (MHz)	Calculated Frequency / (MHz)	Obs.– Calc. / (MHz)
$5 \rightarrow 4$	15838.6220	15838.6280	-0.0011
$4 \rightarrow 3$	12670.9140	12670.9113	0.0027
$3 \rightarrow 2$	9503.1950	9503.1887	0.0063

Supplementary Data Table 24: $^{195}Pt^{13}C^{13}C^{12}C$

$J' \rightarrow J''$	Observed Frequency / (MHz)	Calculated Frequency / (MHz)	Obs.– Calc. / (MHz)
$5 \rightarrow 4$	15521.3490	15521.3490	0.0000

Supplementary Data Table 25: $^{195}Pt^{13}C^{12}C^{13}C$

$J' \rightarrow J''$	Observed Frequency / (MHz)	Calculated Frequency / (MHz)	Obs.– Calc. / (MHz)
$6 \rightarrow 5$	18171.4750	18171.4808	-0.0058
$5 \rightarrow 4$	15142.9170	15142.9130	0.0040
$4 \rightarrow 3$	12114.3420	12114.3384	0.0036

Supplementary Data Table 26: $^{195}Pt^{12}C^{13}C^{13}C$

$J' \rightarrow J''$	Observed Frequency / (MHz)	Calculated Frequency / (MHz)	Obs.– Calc. / (MHz)
$6 \rightarrow 5$	17907.8250	17907.8258	-0.0008
$5 \rightarrow 4$	14923.2000	14923.2000	-0.0000
$4 \rightarrow 3$	11938.5690	11938.5678	0.0012

Supplementary Data Table 27: $^{196}Pt^{12}C^{12}C^{12}C$

$J' \rightarrow J''$	Observed Frequency / (MHz)	Calculated Frequency / (MHz)	Obs.– Calc. / (MHz)
$5 \rightarrow 4$	15907.1250	15907.1348	-0.0098
$4 \rightarrow 3$	12725.7250	12725.7167	0.0083
$3 \rightarrow 2$	9544.2980	9544.2928	0.0052

Supplementary Data Table 28: $^{196}Pt^{13}C^{13}C^{13}C$

$J' \rightarrow J''$	Observed Frequency / (MHz)	Calculated Frequency / (MHz)	Obs.– Calc. / (MHz)
$6 \rightarrow 5$	17814.2250	17814.2276	-0.0026
$5 \rightarrow 4$	14845.2000	14845.2016	-0.0016
$4 \rightarrow 3$	11876.1750	11876.1690	0.0060

Supplementary Data Table 29: $^{196}Pt^{12}C^{12}C^{13}C$

$J' \rightarrow J''$	Observed Frequency / (MHz)	Calculated Frequency / (MHz)	Obs.– Calc. / (MHz)
$6 \rightarrow 5$	18243.4750	18243.4766	-0.0016
$5 \rightarrow 4$	15202.9000	15202.9095	-0.0095
$4 \rightarrow 3$	12162.3500	12162.3357	0.0143

Supplementary Data Table 30: $^{196}Pt^{12}C^{13}C^{12}C$

$J' \rightarrow J''$	Observed Frequency / (MHz)	Calculated Frequency / (MHz)	Obs.– Calc. / (MHz)
$5 \rightarrow 4$	15585.2750	15585.2732	0.0018
$4 \rightarrow 3$	12468.2250	12468.2272	-0.0022

Supplementary Data Table 31: $^{196}Pt^{13}C^{12}C^{12}C$

$J' \to J''$	Observed Frequency / (MHz)	Calculated Frequency / (MHz)	Obs.– Calc. / (MHz)
$5 \to 4$	15827.4000	15827.4079	-0.0079
$4 \to 3$	12661.9500	12661.9352	0.0148
$3 \to 2$	9496.4500	9496.4566	-0.0066

Supplementary Data Table 32: $^{196}Pt^{13}C^{13}C^{12}C$

$J' \to J''$	Observed Frequency / (MHz)	Calculated Frequency / (MHz)	Obs.– Calc. / (MHz)
$5 \to 4$	15510.0500	15510.0629	-0.0129
$4 \to 3$	12408.0750	12408.0588	0.0162

Supplementary Data Table 33: $^{196}Pt^{13}C^{12}C^{13}C$

$J' \to J''$	Observed Frequency / (MHz)	Calculated Frequency / (MHz)	Obs.– Calc. / (MHz)
$6 \to 5$	18158.3000	18158.3103	-0.0103
$5 \to 4$	15131.9500	15131.9376	0.0124

Supplementary Data Table 34: $^{196}Pt^{12}C^{13}C^{13}C$

$J' \to J''$	Observed Frequency / (MHz)	Calculated Frequency / (MHz)	Obs.– Calc. / (MHz)
$6 \to 5$	17894.7270	17894.7369	-0.0099
$5 \to 4$	14912.2980	14912.2926	0.0054
$4 \to 3$	11929.8500	11929.8419	0.0081

Supplementary Data Table 35: $^{198}Pt^{12}C^{12}C^{12}C$

$J' \to J''$	Observed Frequency / (MHz)	Calculated Frequency / (MHz)	Obs.– Calc. / (MHz)
$5 \to 4$	15885.2750	15885.2729	0.0021
$4 \to 3$	12708.2250	12708.2272	-0.0022
$3 \to 2$	9531.1750	9531.1756	-0.0006

Supplementary Data Table 36: $^{198}Pt^{13}C^{13}C^{13}C$

$J' \to J''$	Observed Frequency / (MHz)	Calculated Frequency / (MHz)	Obs.– Calc. / (MHz)
$6 \to 5$	17788.0750	17788.0789	-0.0039
$5 \to 4$	14823.4250	14823.4110	0.0140
$4 \to 3$	11858.7250	11858.7366	-0.0116

Supplementary Data Figure 1 – Frontier orbital energy level diagram for PtC_3 calculated at the HF/aug-cc-pVTZ level of theory.

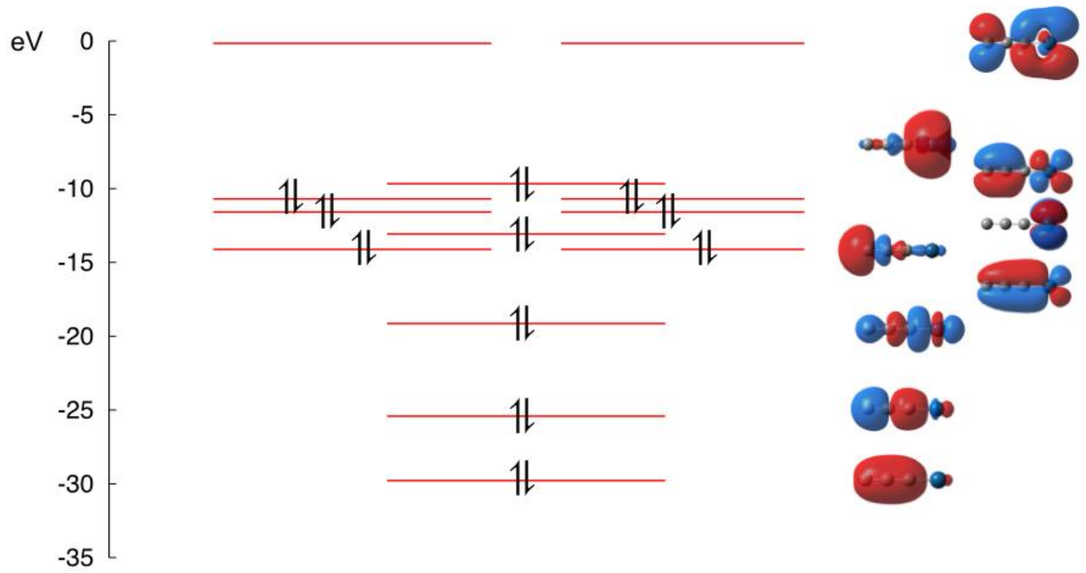

Supplement: Supplementary file 1 — Supplementary [file ANGE-128-3832-s001.pdf]
